# Object-related regularities are processed automatically: evidence from the visual mismatch negativity

**DOI:** 10.3389/fnhum.2013.00259

**Published:** 2013-06-10

**Authors:** Dagmar Müller, Andreas Widmann, Erich Schröger

**Affiliations:** Institut für Psychologie, Universität LeipzigLeipzig, Germany

**Keywords:** deviance detection, human ERP, prediction error, object formation, variable resolution electromagnetic tomography (VARETA), visual mismatch negativity

## Abstract

One of the most challenging tasks of our visual systems is to structure and integrate the enormous amount of incoming information into distinct coherent objects. It is an ongoing debate whether or not the formation of visual objects requires attention. Implicit behavioral measures suggest that object formation can occur for task-irrelevant and unattended visual stimuli. The present study investigated pre-attentive visual object formation by combining implicit behavioral measures and an electrophysiological indicator of pre-attentive visual irregularity detection, the visual mismatch negativity (vMMN) of the event-related potential. Our displays consisted of two symmetrically arranged, task-irrelevant ellipses, the objects. In addition, there were two discs of either high or low luminance presented on the objects, which served as targets. Participants had to indicate whether the targets were of the same or different luminance. In separate conditions, the targets either usually were enclosed in the same object or in two different objects (standards). Occasionally, the regular target-to-object assignment was changed (deviants). That is, standards and deviants were exclusively defined on the basis of the task-irrelevant target-to-object assignment but not on the basis of some feature regularity. Although participants did not notice the regularity nor the occurrence of the deviation in the sequences, task-irrelevant deviations resulted in increased reaction times. Moreover, compared with physically identical standard displays deviating target-to-object assignments elicited a negative potential in the 246–280 ms time window over posterio-temporal electrode positions which was identified as vMMN. With variable resolution electromagnetic tomography (VARETA) object-related vMMN was localized to the inferior temporal gyrus. Our results support the notion that the visual system automatically structures even task-irrelevant aspects of the incoming information into objects.

## Introduction

In everyday life our visual system is challenged with a multitude of information which has to be structured into coherent objects. There is a long-standing debate on whether or not the formation of visual objects requires attention. Evidence supporting the significance of attention for visual object formation for example comes from experiments in which participants searched for targets defined by a conjunction of two features. Reaction times in such experiments typically increase with the number of objects presented on the display thus suggesting that attention had to be shifted serially in order to form feature-conjunctions (Treisman and Gelade, 1980). Moreover, when objects bearing two different features were presented outside the focus of attention participants reported the occurrence of illusory conjunctions, i.e., the combination of features originally belonging to different items (Treisman and Schmidt, 1982). The opposing view, i.e., the approach of pre-attentive or automatic object formation, receives support from studies showing that participants judged two task-relevant features more accurately and/or faster when the features belonged to one object compared with when the features belonged to two different objects overlapping in space (e.g., Duncan, 1984; for a review of similar studies see, Scholl, 2001). Additional evidence for automatic object formation comes from another line of experiments which showed that the processing of centrally presented targets was affected by the organization of task-irrelevant and unattended elements presented in the background (e.g., Driver et al., 2001; Kimchi and Razpurker-Apfeld, 2004; Lamy et al., 2006; Kimchi and Peterson, 2008; Shomstein et al., 2010).

In such behavioral studies automatic object formation solely is indicated by the responses given by the participants. Event-related potentials (ERPs), which can be elicited by task-irrelevant, unattended aspects of the stimulation, may be exploited for investigating automatic object formation as such an approach could shed light on the temporal characteristics of automatic object formation as well as on the related cortical structures. In the auditory modality, several studies used the mismatch negativity (MMN) component to demonstrate automatic grouping of sounds into objects (e.g., Ritter et al., 2000; Atienza et al., 2003; Winkler et al., 2003; Sussman et al., 2007). The MMN is elicited when the actual stimulus deviates from a prediction generated on the basis of some regularity inherent to the preceding stimulus sequence (for a recent review see, Näätänen et al., 2011). In the past two decades it has been shown that there is an analogue mechanism extracting regularities from the visual environment and thus, generating predictions upon upcoming visual stimuli (for reviews see, Pazo-Alvarez et al., 2003; Czigler, 2007; Kimura et al., 2011b). If the actual input features an irregularity and thus mismatches the predicted stimulus a prediction error occurs which is thought to be reflected by the visual mismatch negativity (vMMN) component (Kimura et al., 2011b; Winkler and Czigler, 2012). It was convincingly shown that this mechanism operates in an automatic manner. That is, regularities are extracted irrespective of that they are not relevant for the task at hand and even when any possible intentional processing is prevented by masking (Kogai et al., 2011) or by presenting irregularities within the time window of the “attentional blink” (Berti, 2011). Recent studies have shown that this automatic system is capable of indicating not only highly salient violations of feature-regularities (e.g., a red-colored stimulus within a sequence of green-colored stimuli) but also less salient violations of regularities related to feature conjunctions (Winkler et al., 2005), facial emotional expressions (e.g., Astikainen and Hietanen, 2009; Chang et al., 2010; Kimura et al., 2011a; Stefanics et al., 2012), vertical mirror symmetry (Kecskes-Kovacs et al., 2013) or hand laterality (Stefanics and Czigler, 2012).

**Figure 1:**
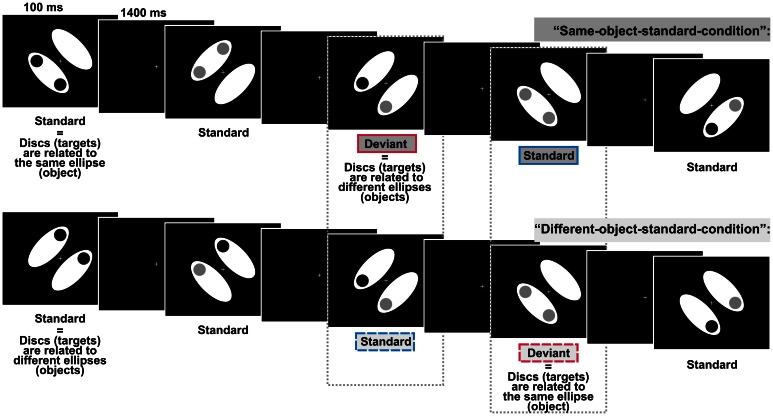
**Schematic display sequences presented in the two experimental conditions defined by comprising different object-related regularities**. In both conditions, regularly presented standard displays and irregularly presented deviant displays differed only with respect to the relation between the discs and the ellipses (the objects), i.e., discs could either belong to the same ellipse or to different ellipses. Participants had to evaluate whether the two discs (the targets) were of the same or of different luminance whereas the target-to-object relation was task-irrelevant. Dashed boxes indicate, that we compared the processing of physically identically displays, i.e., we compared deviants from the “different-object-standard-condition” with standards from the “same-object-standard-condition” (right box) and deviants from the “same-object-standard-condition” with standards from the “different-object-standard-condition” (left box).

In the present study we investigated whether task-irrelevant violations of the regular assignment of single elements into visual objects elicited the vMMN. The elicitation of vMMN would indicate that the formation of visual objects can take place automatically which would make an important contribution to the aforementioned debate on the role of attention in visual object formation. In a previous study we could show that the automatic visual regularity detection system indexed by the vMMN is sensitive to object information: task-irrelevant color-irregularities were processed differently when the irregularities belonged to the same object compared with when they belonged to different objects (Müller et al., 2010), thus supporting automatic object formation by an electrophysiological measure. However, it is critically noteworthy that the highly salient color-irregularities used in this design could have induced involuntary attention shifts toward the task-irrelevant objects (e.g., Hopfinger and Mangun, 2001; Theeuwes, 2004). Thus, we designed the present experiment to rule out that object-specific processing is contingent on the processing of salient irregularities. Our displays consisted of two symmetrically arranged, task-irrelevant ellipses, the objects. In addition, there were two task-relevant discs of either high or low luminance presented on the objects. Thus, each of the ellipses and the discs presented on it should be combined to a common object based on the Gestalt principle of common region (Palmer, 1992). Participants had to judge the luminance of the discs (same vs. different, *p* = 0.5, respectively). We investigated object-related processing by varying the assignment of task-irrelevant objects and task-relevant discs. Frequently presented standard displays were characterized by a regular disc-to-object assignment, i.e., in two separate conditions regularly the discs either belonged to the same object or to different objects. In contrast, occasionally occurring deviant displays (*p* = 0.125) were characterized by a non-salient change in the regular disc-to-object assignment (see Figure 1 for illustration). That is, standard displays and deviant displays consisted of the same elements, but differed only with regard to the task-irrelevant disc-to-object assignment. If in such a design deviant displays indeed elicit the vMMN we can draw a twofold conclusion: (1) As regularities and irregularities in our design are solely defined by object-related characteristics deviant displays will elicit the vMMN only if object-related information is encoded before the irregularity detection system checks the actual input, i.e., the elicitation of vMMN would support the notion of automatic object formation. (2) As standard displays and deviant displays in our design are not confounded by physical differences the elicitation of vMMN would show that the automatic visual irregularity detection system is not restricted to the detection of salient lower-order irregularities based on physical differences between standards and deviants but is also sensitive to the detection of non-salient higher-order irregularities.

## Material and methods

### Participants

Sixteen healthy students (10 women and 6 men, aged 18–30 years, mean age = 24.9 years) participated in the experiment for either course credit or payment. All of them reported normal or corrected-to-normal vision. Written informed consent was obtained from all of them according to the ethical code of the World Medical Association (Declaration of Helsinki). Data of two additional participants were excluded due to excessive eye movements which resulted in rejecting more than 50% of the trials from EEG analysis.

### Stimuli and procedure

Stimulus presentation and the collection of behavioral responses were realized using the MATLAB toolbox Cogent2000v1.28. Stimuli were presented on a 19″ color monitor (ViewSonic Graphics Series G90fB) set at a resolution of 1024 × 768 with a refresh rate of 100 Hz. We used a chinrest to maintain the viewing distance at 50 cm. Each test display consisted of two white ellipses (each subtending a visual angle of 7.97 × 3.43°, 148.3 cd/m^2^), two discs (diameter 1.72°), and a centrally presented white fixation cross (0.57 × 0.57°). Ellipses were arranged in parallel and flanked the fixation cross. The distance between the center of each ellipse and the center of the display was 2.52°. In different displays ellipses were pseudo-randomly tilted 45° either to the left or to the right in relation to the vertical midline. Displays containing left- and right-tilted ellipses occurred equiprobably within each block. In the following ellipses will be referred to as the “objects.” The two discs were presented equally likely at two adjacent out of four possible positions (up, low, left, right, each 3.43° off the display-center) and were either of low luminance (dark-gray, 14.55 cd/m^2^) or high luminance (light-gray, 80.6 cd/m^2^). In different displays the two discs were of either the same luminance (i.e., both discs were either dark-gray or light-gray) or different luminance (i.e., one disc was dark-gray and the other light-gray). Displays containing discs of the same luminance vs. different luminance occurred equiprobably within each block. As the luminance of the discs was task-relevant discs will be referred to as targets. In two separate experimental conditions we varied the target-to-object assignment: usually (*P* = 0.875) the targets were presented on either the same object (standards of “same-object-standard-condition”) or on different objects (standards of “different-object-standard-condition”). Occasionally and unpredictably (*P* = 0.125), the regular assignment of the targets to the objects was exchanged: targets were presented on either different objects (deviants of “same-object-standard-condition”) or on the same object (deviants of “different-object-standard-condition”). That is, deviants were exclusively defined on the violation of the regular target-to-object assignment whereas there were no physical differences between standard- and deviant-displays. All stimuli were presented against a black background. The fixation cross was shown constantly throughout a block (see Figure 1 for an illustration of the design).

Each test-display was shown for 100 ms and was followed by an inter-stimulus interval of 1400 ms. Standard and deviant displays were presented in randomized order with the restriction that deviant-displays were always followed by at least two standard-displays. Stimuli were delivered in blocks of 128 trials each. The experiment consisted of 8 blocks of the “same-object-standard-condition” and 8 blocks of the “different-object-standard-condition,” respectively. Blocks were presented in pseudo-randomized order with the restriction that four blocks of each condition were included in the first and second half of the experiment, respectively. Including individual breaks between the blocks the experiment lasted about 1 h.

Participants were instructed to indicate as fast and as accurate as possible whether the two discs presented in each test display had the same or different luminance, i.e., disc-luminance was task-relevant whereas the disc-to-object assignment defining deviant- and standard-displays was task-irrelevant. Responses were given by pressing the outermost left/right button of a 4-button response pad with the left/right index finger. Response-to-button assignment (i.e., same/different luminance required left/right button presses and vice versa) was changed after completing the first half of experimental blocks. Subjects completed a training block of 32 trials in order to become acquainted with the task. In contrast to the experimental blocks in the training block the duration of test displays was increased to 300 ms. At the end of each block participants got feedback on their performance (mean reaction times and number of incorrect responses). We motivated the participants to focus on the task by rewarding each block in which they reached a certain criterion (not exceeding five incorrect responses, i.e., a hit rate of 96.1% minimum) with paying 25 cent. In addition, the participant with the highest performance received a book token of 10 Euro value.

After completing the experiment, we asked the subjects whether they noticed something specific in the design of the experiment. If they did not comment on the relation between task-relevant discs and objects by themselves we explicitly asked whether the realized that there was a “default” target-to-object assignment within each block which infrequently changed.

### Electrophysiological recording

The electroencephalogram (EEG) was recorded continuously with a BrainAmp amplifier system (Brain Products GmbH, Munich, Germany) from 60 active electrodes mounted into an elastic cap according to the extended international 10–20 system (Fp1, FP2, AF3, AF4, F7, F5, F3, F1, Fz, F2, F4, F6, F8, FT7, FC5, FC3, FC1, FC2, FC4, FC6, FT8, T7, C5, C3, C1, Cz, C2, C4, C6, T8, TP9, TP7, CP5, CP3, CP1, CPz, CP2, CP4, CP6, TP8, TP10, P/, P5, P3, P1, Pz, P2, P4, P6, P8, PO9, PO7, PO3, POz, PO4, PO8, PO10, O1, Oz, O2). Horizontal and vertical eye movements were monitored by electrodes placed at the outer canthi of both eyes and above (electrode at position Fp2 was used) and below the right eye, respectively (electro-oculogram, EOG). An electrode attached at the tip of the nose served as off-line reference. Additional active electrodes placed at position FCz and AFz served as on-line reference and ground electrode, respectively. Data were filtered online (0.1–250 Hz bandpass) and sampled at 500 Hz.

### Analysis of behavioral data

We calculated mean reaction times (RTs) and mean hit rates separately for the two stimulus types (standards vs. deviants) and the two target-to-object assignments (discs in the same object vs. discs in different objects). For the calculation of mean RTs, RTs related to incorrect responses and RTs out of a range individually defined by the mean RT calculated from all correct responses ± 2 standard deviations were excluded. Both RTs and hit rates were subjected to repeated measures ANOVAs with the factors of STIMULUS TYPE and TARGET-TO-OBJECT ASSIGNMENT, i.e., we compared responses given to physically identically deviant- and standard-stimuli obtained across the two different experimental conditions (see also Figure 1 for an illustration of the comparisons).

### Analysis of electrophysiological data

Offline, EEG activity was re-referenced to the activity recorded from an electrode placed at the tip of the nose, and EEG and EOG activity was filtered (0.5–40 Hz band-pass digital FIR filter with a length of 1025 points). EEG and EOG activity was epoched from −100 ms before to 700 ms after the onset of test displays. The first 100 ms of each epoch served as the baseline interval. Epochs containing signal changes exceeding 100 μV at any electrode, epochs related to displays to which participants did not respond (misses) or responded incorrectly (mistakes), epochs immediately following misses and mistakes and epochs related to standard displays directly following a deviant display were excluded from further analysis. Epochs were averaged separately for standards and deviants presented in the “same-object-standard-condition” and in the “different-object-standard-condition,” respectively. On average (mean ± SD), there were 586 ± 50/99 ± 7 epochs for standards/deviants from the “same-object-standard-condition” and 577 ± 70/97 ± 13 epochs for standards/deviants from the “different-object-standard-condition” available for each participant.

To analyse genuine deviant-specific ERP responses, we calculated difference waves by subtracting ERPs elicited by standard displays from those elicited by physically identically deviant displays (i.e., standard-ERPs from the “same-object-standard-condition” were subtracted from deviant-ERPs from the “different-object-standard-condition” and standard-ERPs from the “different-object-standard-condition” were subtracted from deviant-ERPs from the “same-object-standard-condition”).

Visual inspection revealed that deviant and standard ERPs differed prominently at posterio-temporal electrode sites at about 260 ms latency, i.e., in the N2 latency range. Accordingly, we determined individual N2 peak latencies at electrode sites P5/6, P7/8, and PO7/8 in the 230–290 ms time range separately for each stimulus type (standard vs. deviant) and each target-to-object assignment (discs in the same object vs. discs in different objects). As the N2 peaked slightly earlier in trials in which discs belonged to the same object compared with trials in which discs belonged to different objects [main effect of factor TARGET-TO-OBJECT ASSIGNMENT, *F*_(1, 15)_ = 7.28, *p* = 0.017, η^2^_*p*_ = 0.33] we adapted the position of 30-ms time windows used for computing individual mean amplitudes accordingly (246–276 ms/250–280 ms for trials in which discs belonged to the same object/to different objects). Additionally to the posterio-temporal region of interest (ROI) which comprises of the collapsed mean amplitudes at P5/7, P7/8, PO7/8, we selected a frontal ROI (AF3/4, F3/4, F5/6) to check for the occurrence of frontal deviant-related effects (Czigler et al., 2002). We tested for the significance of differences between standard- and deviant-responses by conducting a repeated measures ANOVA with the factors of STIMULUS TYPE × TARGET-TO-OBJECT ASSIGNMENT × HEMISPHERE (left vs. right) × ROI (posterio-temporal vs. frontal). Follow-up analyses comparing standard- and deviant-responses separately for the left and the right hemisphere and the two ROIs were carried out by paired, two-tailed Student's *t*-tests. The alpha level criterion for all statistical analyses was set to.05. Effect sizes are presented as partial eta square (η^2^_*p*_).

We plotted voltage topography and scalp current density (SCD) maps of ERPs elicited by deviants and standards, and of the deviant-minus-standard difference potentials. Calculation and plotting was carried out by using the *sphspline* plug-in (Widmann, 2006) for EEGlab (Delorme and Makeig, 2004). As there were no striking differences in the distribution of deviant-related activity between the two target-to-object assignments we collapsed the data obtained in the two conditions. The time window was set to 246–280 ms thus, equally comprising the peaks of deviant-related activity of both target-to-object assignments. Furthermore, we applied Variable Resolution Electromagnetic Tomography (VARETA, Bosch-Bayard et al., 2001) in order to localize cortical generators of deviant-related activity. The VARETA technique uses a discrete spline distributed inverse model to estimate the spatially smoothest intracranial distribution of primary current densities that correspond to the EEG-signals measured at the scalp. In doing so VARETA estimates the smoothing parameter voxel-wise, thus allowing for variable amounts of spatial smoothness and localizing discrete and distributed sources with equal accuracy (Bosch-Bayard et al., 2001; Pizzagalli, 2007). We mapped possible sources on a 3D regular grid model (3244 voxels, 7 mm grid spacing) based on the probabilistic brain tissue maps available from the Montreal Neurological Institute (MNI, Evans et al., 1993) which restricts sources to the gray matter. Significant activations were displayed as 3D-images by computing statistical parametric maps of the estimated primary current densities based on a voxel-by-voxel Hoteling *T*^2^-test against zero. Random field theory (Worsley et al., 1996) was applied to correct thresholds for spatial dependencies between voxels. To localize deviant-specific activation we contrasted the solutions obtained for deviants with those obtained for standards.

## Results

### Behavioral data

When we asked for specifics of the design at the end of the experiment five out of our 16 participants reported that the task-relevant discs and the enclosing ellipses (i.e., the objects) were somehow related: they noticed that the targets could be enclosed in either the same object or in different objects. However, all but one^1^ did not report spontaneously that they realized any difference in the frequency of the occurrence of the two types of target-to-object assignments. Even after we presented a figure displaying both target-to-objects assignments and we explicitly inquired whether they occurred with different frequencies none of the participants reported that they realized the occurrence of frequently and infrequently presented assignments within one block, i.e., participants neither realized object-based regularities nor violations of these regularities.

However, results of the repeated measures ANOVA with the factors STIMULUS TYPE (standards vs. deviants) and TARGET-TO-OBJECT ASSIGNMENT (discs belonging to the same object vs. discs belonging to different objects) conducted on the reaction times showed that the performance of the participants was significantly influenced by the (unnoticed) object-based regularities: participants responded significantly faster in trials with frequently presented target-to-object assignments (i.e., in standard trials, mean RT 505 ms ± 14 ms *SEM*) compared with trials with infrequent target-to-object assignments [i.e., in deviant trials, 515 ± 14 ms, main effect of factor STIMULUS TYPE: *F*_(1, 15)_ = 35.5, *p* < 0.001, η^2^_*p*_ = 0.70]. Furthermore, participants responded slightly faster when discs belonged to different objects compared with when discs belonged to the same object [507 ± 14 ms vs. 513 ± 14 ms, main effect of factor TARGET-TO-OBJECT ASSIGNMENT: *F*_(1, 15)_ = 5.75, *p* = 0.03, η^2^_*p*_ = 0.28]. There was no interaction of the two factors [*F*_(1, 15)_ = 0.35, *p* > 0.5]. On average participants responded correctly in 95.76% ± 0.5 of all trials. Hits rates were not significantly affected by neither the factor STIMULUS TYPE nor TARGET-TO-OBJECT ASSIGNMENT (both *F* < 1). The interaction of the two factors only marginally failed to reach significance [*F*_(1, 15)_ = 4.52, *p* = 0.051, η^2^_*p*_ = 0.23]. However, none of the possible follow-up comparisons reached significance [all *t*_(df = 15)_ < −1.65, all *p* > 0.1 even without correction for multiple comparisons]. Behavioral results are summarized in Table 1.

**Table 1:**
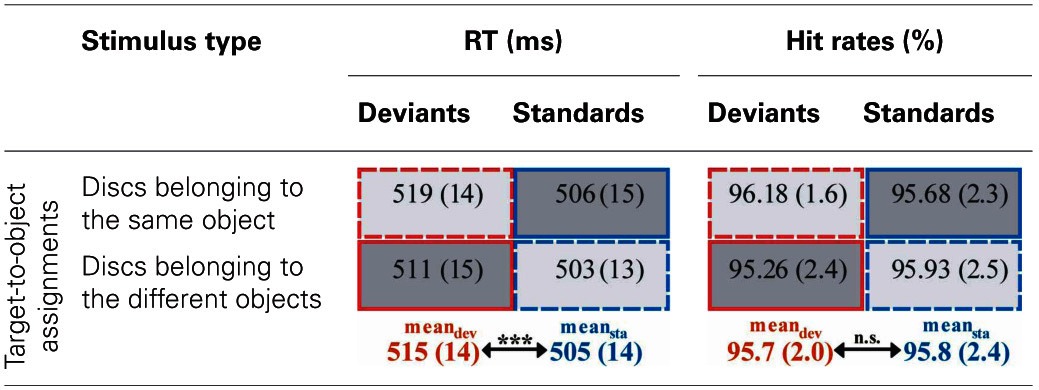
**Behavioral performance**.

Reaction times (RT) and hit rates are displayed separately for deviant (red outlines) and standard trials (blue outlines) for the two target-to-object assignments, respectively. SEM are given in parentheses. Cells containing responses given within one experimental condition are marked by identical gray-scale and line-style (dark-gray cells with solid outlines correspond to the “same-object-standard-condition,” light-gray cells with dashed outlines correspond to the “different-object-standard-condition”). Responses given to physically identically deviants and standards are contrasted line-by-line. Asterisks indicate significant differences between deviant- and standard-responses averaged over the two target-to-object assignments (^***^p < 0.001).

### Electrophysiological data

**Figure 2:**
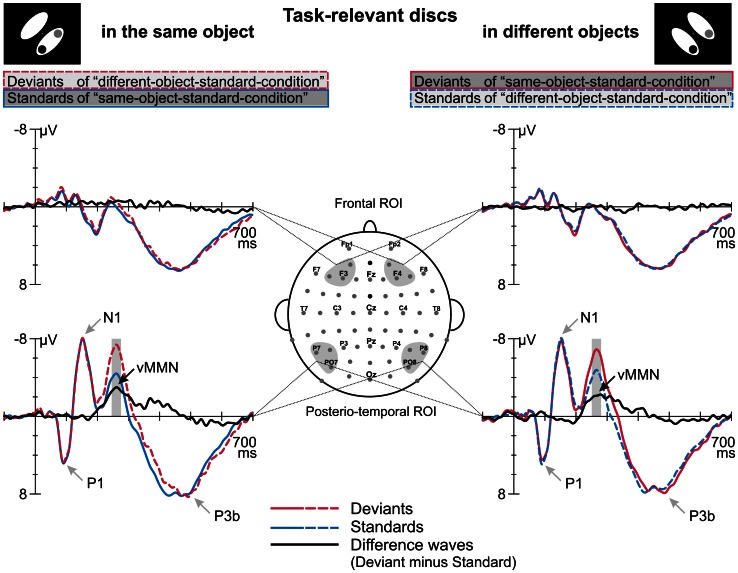
**Event-related potentials elicited by deviants and standards which were defined by irregular and regular target-to-object assignments, respectively, and the corresponding deviant minus standard difference waves**. ERPs and difference waves are displayed separately for the two target-to-object assignments (left column, discs belonging to the same object; right column, discs belonging to different objects). We found differences in the processing of standards and deviants at a posterior-temporal region of interest (ROI, lower row) whereas no such differences occurred at frontal electrode positions (frontal ROI, upper row). Gray shaded boxes indicate the time windows used to determine mean amplitudes which were subjected to statistical analysis. The peaks of prominent ERP components are indicated by gray arrows.

**Figure 3:**
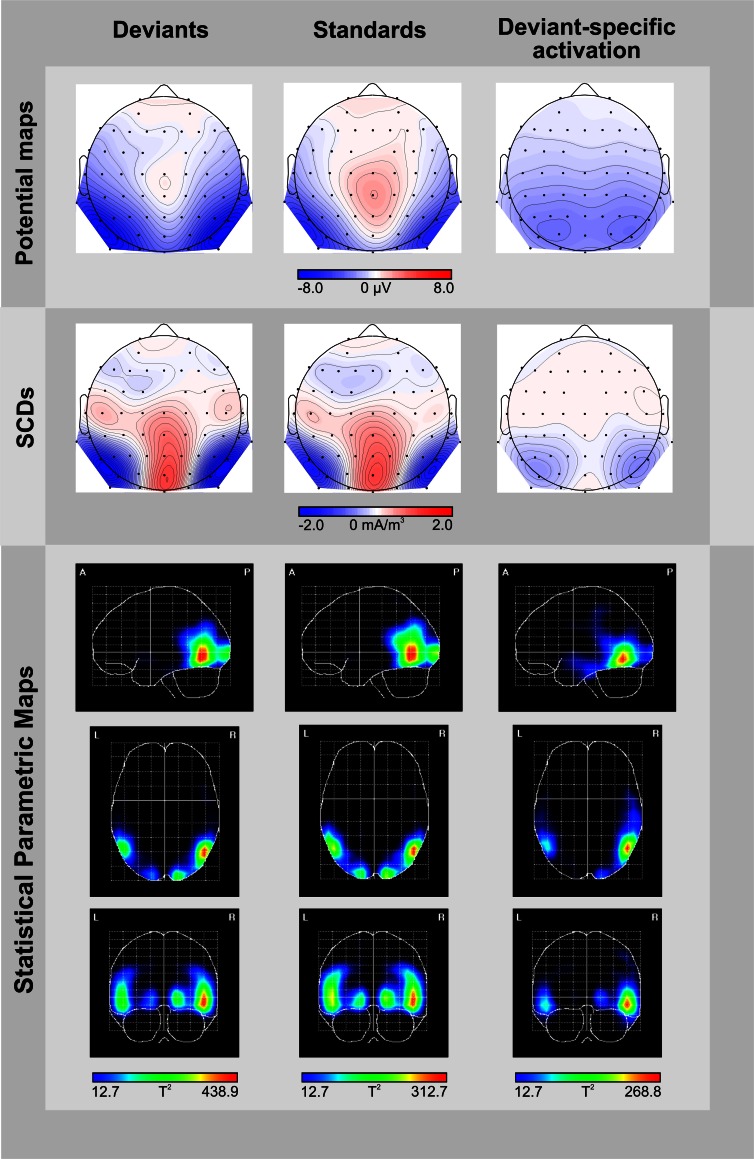
**Topographic and tomographic distribution of ERP-responses elicited by deviants (left column) and standards (middle column), and the corresponding deviant-specific activity (right column) in the 246–280 ms time window**. Potential maps (first row) and scalp current density maps (SCDs, second row) are shown with a distance of 0.5 μV and 0.1 mA/m^3^ between isocontour lines, respectively. To display deviant-specific activity potential maps and SCDs were calculated for the deviant-minus-standard difference waves. A smoothing parameter of lambda = 10^−5^ was applied to the SCDs. Source localizations computed by VARETA are displayed as statistical parametric maps (third row), thus illustrating the probability of activation within cortical regions (threshold *T*^2^ > 12.7 corresponds to a Bonferroni-corrected *p* < 0.0001). Deviant-specific source localization is displayed as the contrast between the solutions obtained for deviants vs. standards.

Figure 2 displays the grand average ERPs elicited by deviant and standard displays superimposed with the respective deviant-minus-standard differences waveforms, separately for the two target-to-object assignments (discs belonging to the same object vs. discs belonging to different objects). Deviant and standard displays of both target-to-object assignments elicited a representative sequence of prominent visual ERP components at posterior electrode sites: P1 peaking at 95 ms, N1 at 150 ms, P2 at 205 ms and N2 at around 260 ms which was followed by a broad-peaked P3b in the 350–550 ms latency range (Figure 2). In the P1 and N1 latency range deviant and standard ERPs are nearly perfectly matched. In contrast, in the N2 latency range deviant ERPs clearly show a more negative response than standard ERPs. Visual inspection revealed that deviant-specific responses were most prominent at posterior-temporal electrode sites (Figure 2, lower row) whereas there were no deviant-specific responses at frontal electrode sites (Figure 2, upper row). The posterio-temporal distribution of deviant-specific responses is also illustrated by the corresponding potential maps and SCD maps (Figure 3, upper and middle row). Visual inspection further revealed that there were no differences between deviant and standard ERPs at fronto-central electrode sites at latency ranges around 400 ms post-stimulus, i.e., we did not find evidence that deviants elicit the P3a component.

Results of a repeated measures ANOVA conducted on the mean amplitudes in the N2 latency range with the factors STIMULUS TYPE (standards vs. deviants) × TARGET-TO-OBJECT ASSIGNMENT (discs in the same object vs. discs in different objects) × HEMISPHERE (left vs. right) × ROI (posterio-temporal vs. frontal) confirmed that deviants exhibited significantly more negative amplitudes than standards [main effect of STIMULUS TYPE: *F*_(1, 15)_ = 28.19, *p* < 0.001, η^2^_*p*_ = 0.65]. This effect was restricted to the posterior-temporal ROI [interaction of STIMULUS TYPE × ROI, *F*_(1, 15)_ = 53.59, *p* < 0.001, η^2^_*p*_ = 0.78]. A significant threefold interaction of STIMULUS TYPE × HEMISPHERE × ROI [*F*_(1, 15)_ = 5.56, *p* = 0.032, η^2^_*p*_ = 0.27] suggests that deviant specific responses found at the posterior-temporal ROI were more accentuated in the right hemisphere (−2.6 μV ± 0.4 SEM vs. −2.3 μV ± 0.4 in the right vs. left hemisphere). Follow-up analyses, however, failed to reach significance [*t*_(df = 15)_ = −1.67, *p* > 0.1]. In general, amplitudes at the posterior-temporal ROI were more negative than amplitudes at the frontal ROI [main effect of ROI, *F*_(1, 15)_ = 20.58, *p* < 0.001, η^2^_*p*_ = 0.58]. Amplitudes were not modulated by neither the TARGET-TO-OBJECT ASSIGNMENT [*F*_(1, 15)_ = 3.14, *p* = 0.1] and the HEMISPHERE [*F*_(1, 15)_ = 0.03, *p* = 0.87] itself nor by anyone of the other possible interactions of factors [all *F*_(1, 15)_ < 3.25, all *p* > 0.09]. Mean amplitudes of deviant and standard responses for the two target-to-object assignments are summarized separately for the posterior-temporal ROI and the frontal ROI, respectively, in Table 2.

**Table 2:**
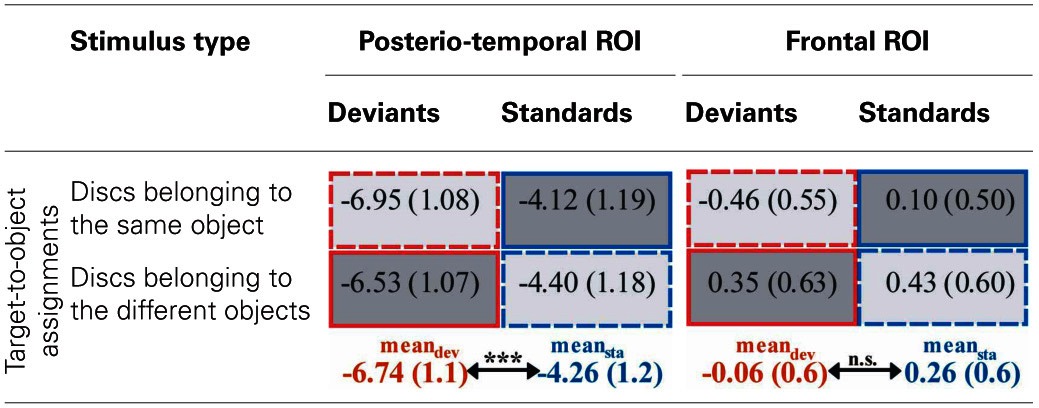
**Mean amplitudes (μV) elicited by deviants (red outlines) and standards (blue outlines) at posterio-temporal ROI (electrodes P5/6, P7/8, PO7/8) and frontal ROI (electrodes AF3/4, F3/4, F5/6) in the N2-latency range**.

Responses are displayed separately for the two target-to-object assignments. SEM are given in parentheses. As in Table 1 cells containing responses given within one experimental condition are marked by identical gray-scale and line-style (dark-gray cells with solid outlines correspond to the “same-object-standard-condition,” light-gray cells with dashed outlines correspond to the “different-object-standard-condition”). Responses given to physically identically deviants and standards are contrasted line-by-line. Asterisks indicate significant differences between deviant- and standard-responses averaged over the two Target-to-object assignments (^***^p < 0.001).

Numerically the vMMN-amplitudes differed between the two target-to-object assignments (−2.83 ± 0.4 μV vs. −2.13 ± 0.4 μV when discs belonged to the same vs. different objects). However, within the present data this difference does not reach significance [interaction between the factors STIMULUS TYPE × TARGET-TO-OBJECT ASSIGNMENT F_(1, 15)_ = 0.3, p = 0.1 when we conducted the ANOVA for the posterior ROI only].

The potential map of the deviant-minus-standard difference waves reveals a broadly distributed occipito-temporal two-peaked negative potential (Figure 3, upper row, right column). The corresponding SCD topography exhibits prominent bilateral occipito-temporal sinks accompanied by a weak source over the central occipital region and distributed weak sources over fronto-central areas (Figure 3, middle row, right column). Source analyses conducted by the VARETA approach show that brain activity elicited by deviant-trials is generated in the posterior part of the inferior/middle temporal gyrus (MNI coordinates *X*, *Y*, *Z*: 50/−50, −62, −10) and at the occipital pole (17/−17, −95, −1, Figure 3, lower row, left column). Activity elicited by standard-trials is generated more superiorly in the middle temporal gyrus (50/−50, −62, −2) and at the occipital pole (15/−15, −98, −2), too (Figure 3, lower row, middle column). We contrasted source localizations obtained for deviants and standards for highlighting regions with deviant-specific activation. Deviant-specific activation was generated in the inferior temporal gyrus (50/−50, −62, −10, Figure 3, lower row, right column) and showed a right-hemispheric accentuation.

## Discussion

In the present study, we investigated automatic visual object formation by testing whether task-irrelevant violations of object-based regularities are capable of (1) influencing implicit behavioral measures and (2) eliciting the vMMN—an automatic ERP-component which indexes the detection of a mismatch between the actual stimulus and a prediction generated on the basis of regularities extracted from the preceding sequence of stimuli (Kimura et al., 2011b). Importantly, in the present design violations of object-based regularities were exclusively related to the (non-salient) assignment of task-relevant elements of the display to the objects, i.e., there were no salient violations of feature-regularities. Indeed, our participants did not notice any object-related regularity or any violation of regularity within the sequence of stimuli. Nevertheless, task-irrelevant violations of object-related regularities resulted in increased reaction times. This result is in line with behavioral studies which showed that the regular organization of task-irrelevant background elements influences the processing of task-relevant items via perceptual grouping (e.g., Driver et al., 2001; Kimchi and Razpurker-Apfeld, 2004; Russell and Driver, 2005; Lamy et al., 2006; Kimchi and Peterson, 2008; Shomstein et al., 2010).

Extending these behavioral indicators, our study provides electrophysiological evidence for automatic visual object formation: compared with physically identical standard displays, those displays violating the regular target-to-object assignment elicited higher negative potentials in the 246–280 ms time window over posterio-temporal electrode positions. Latency and topography of this negative difference potential correspond to the characteristics of the vMMN elicited in an experiment designed to disentangle effects of sensory, N1-refractoriness-based deviance detection from genuine cognitive effects based on the violation of predictions (Kimura et al., 2009). Our results show that in the P1-N1 latency range ERPs elicited by deviant- vs. standard-displays were nearly perfectly matched. Thus, we could convincingly show that the visual system is capable of detecting violations of higher-level regularities automatically even if those violations are not accompanied by N1-refractoriness-effects. Moreover, as we did not find any evidence for the elicitation of the P3a component—a component which is considered as an indicator of involuntary attention shifts (for a review see, Escera et al., 2000)—we conclude that the irregularities in our design indeed were detected without shifting attention toward the task-irrelevant aspects of the displays. In contrast, in visual studies investigating the processing of salient lower-order regularities/irregularities the elicitation of N1-differences/vMMN was accompanied by the elicitation of the P3a. For this reason in those studies the behavioral impairment observed in the processing of irregular stimuli was ascribed to costs related to involuntary attention shifts toward task-irrelevant aspects of the stimuli (Berti and Schröger, 2001, 2004, 2006; Kimura et al., 2008a,b). In contrast, in our design we observed increased reaction times for irregular displays compared with regular displays without an accompanying P3a (for similar results obtained in a visual multi-deviant design see, Grimm et al., 2009). Thus, the differences in the reaction times could be (at least partly) due to the facilitated processing of regular displays rather than the exclusive impaired processing of irregular displays. However, the elicitation of vMMN in our design suggests that the processing of irregular displays was associated with genuine costs, too.

So far, the automatic detection of higher-level regularities in the visual modality was shown by means of facial emotional expressions only [reviewed in Winkler and Czigler (2012)]. Our results show that the detection of higher-level regularities is not restricted to the ecologically highly important emotional expression of human faces but extends to rather general element-to-object assignments as regularities and irregularities in our design were solely defined on the basis of object-related characteristics. The elicitation of vMMN by object-related irregularities suggests that the process of object formation must have preceded the process of irregularity detection, i.e., our results support the notion of automatic visual object formation based on the Gestalt principle of common region. This conclusion fits to a recently published article reporting the elicitation of vMMN by violations of a conditional rule: task-irrelevant stimuli were presented pairwise in close temporal proximity with regularly both stimuli within one pair had the same color whereas irregularly the second stimulus of a pair took on a different color (Stefanics et al., 2011). As both colors occurred equiprobably within one experimental block regularities/irregularities were defined on the basis of the relation between the two elements of a pair (e.g., if the first stimulus is green then the second stimulus is green, too). Pairs of stimuli in this design can be seen as objects based on the Gestalt principle of temporal proximity. Thus, as in our study, object formation must have preceded the process of irregularity detection which suggests automatic visual object formation to be a more general mechanism. Such automatically formed object representations were recently suggested to be regarded as components of generative models which on the one hand predict the specifics of the upcoming stimulation and which on the other hand are modified by mismatches between the predicted and the actual stimulus (Winkler and Czigler, 2012).

In our study, we identified brain structures related to the violation of object-based regularities by computing SCD maps and applying VARETA. Our SCD maps show a bilateral occipital/occipito-temporal distribution of deviant-specific negative potentials in the 246–280 ms time range. Source analysis carried out by the VARETA technique localized our object-related vMMN to the posterior part of the inferior temporal gyrus (Brodmann's area 37). In numerous articles the inferior temporal gyrus—a structure belonging to the ventral pathway of visual information processing- was shown to be associated with high-order visual object processing in humans or macaques (e.g., Baizer et al., 1991; Goodale and Milner, 1992; Malach et al., 1995; Ishai et al., 1999; Haxby et al., 2001; Grill-Spector and Malach, 2004). The localization of object-related effects of irregularity detection in the inferior temporal gyrus corroborates our recently published localization data (Müller et al., 2012). Here, a combination of object-related and feature (color)-related irregularities generated activation in the inferior temporal gyrus, too. Moreover, also the aforementioned vMMN studies on facial emotional expressions, i.e., on material containing higher-order regularities, found activation related to regularity violation in the inferior temporal gyrus (Kimura et al., 2011a; Stefanics et al., 2012). In contrast, vMMN studies based on feature (orientation and/or color)-related regularities localized deviant-specific activity to earlier anatomical structures of the cortical visual system (occipital lobe—BA 19—Kimura et al., 2010; middle occipital gyrus—Urakawa et al., 2010a,b; occipital fusiform regions—BA 17, 18, 19/7—Yucel et al., 2007). The activation of different feature-/stimulus-specific cortical structures by different types of deviants parallels results from irregularity detection in the auditory modality (e.g., Alain et al., 1999; Rosburg, 2003; Grimm et al., 2006). Interestingly, in all of the vMMN studies cited above deviant-specific activity based on higher-order irregularities or on feature irregularities was additionally found in prefrontal cortical regions (mainly the inferior frontal/medial frontal cortex). It seems plausible to assume that this deviant-specific prefrontal activation indicates involuntary attention shifts toward ecologically relevant irregularities in either facial expression (Kimura et al., 2011a; Stefanics et al., 2012) or hand laterality (Stefanics et al., 2012) and toward salient feature irregularities, respectively (Kimura et al., 2010; Urakawa et al., 2010a,b; Yucel et al., 2007). In contrast, the source localization of our non-salient object-related irregularities does not show a prefrontal activation, which might again underline that in our design there were no involuntary attentional shift and object-related information indeed was processed automatically. However, there are alternative suggestions regarding the functional role of the frontal generator of the auditory MMN (1) sensitivity tuning for irregularity detection in the auditory modality (e.g., Doeller et al., 2003), (2) inhibiting the tendency to respond to task-irrelevant auditory irregularities (Rinne et al., 2005), or (3) updating predictive models on the nature of upcoming stimuli (e.g., Garrido et al., 2009). The latter alternative is also taken into account for explaining the function of the frontal generators of the vMMN (Kimura et al., 2011a). The vMMN studies reporting combined cortical activation of feature-/stimulus-specific regions as well as of frontal regions suggest that the detection of irregularities in both the visual and the auditory modality works in a comparable hierarchically organized manner (for a model see Garrido et al., 2009). In contrast, our results as well as several studies on irregularity detection in the auditory modality suggest that irregular stimuli can elicit a mismatch response even without an accompanying frontal activation (for a review on the frontal generator of the auditory MMN see, Deouell, 2007). It remains a topic of further studies to investigate under which conditions irregularity detection is indicated by both stimulus-specific and frontal activation.

In conclusion, our results show (1) that object-based irregularities are automatically detected presumably by the visual subsystem encoding and/or processing object-related information. That is, we showed that object formation based on the Gestalt principle of common region must have occurred before the visual input was checked for the occurrence of regularities/irregularities. As the visual regularity extraction process was shown to work automatically (Berti, 2011; Kogai et al., 2011) we concluded that the process of object formation which in our design necessarily preceded the regularity extraction process should work automatically, too. Thus, our results support the notion of automatic visual object formation which parallels findings from the auditory modality for which the occurrence of automatic object formation also has been proved (e.g., Ritter et al., 2000; Atienza et al., 2003; Winkler et al., 2003; Sussman et al., 2007). (2) Although closely connected to our first conclusion we can state additionally that the detection of irregularities within sequences of visual stimuli is not restricted to salient stimulus attributes but also works for non-salient higher-order stimulus attributes thus emphasizing the sensitivity of processes extracting regularities from our environment.

### Conflict of interest statement

The authors declare that the research was conducted in the absence of any commercial or financial relationships that could be construed as a potential conflict of interest.

## Footnotes

^1^One participant reported that displays containing discs belonging to the same object occurred more frequently throughout the whole experimental session.
